# Adipose-derived stromal cells increase the formation of collagens through paracrine and juxtacrine mechanisms in a fibroblast co-culture model utilizing macromolecular crowding

**DOI:** 10.1186/s13287-022-02923-y

**Published:** 2022-06-11

**Authors:** Rebekka Harary Søndergaard, Lisbeth Drozd Højgaard, Alexander Lynge Reese-Petersen, Cecilie Hoeeg, Anders Bruun Mathiasen, Mandana Haack-Sørensen, Bjarke Follin, Federica Genovese, Jens Kastrup, Morten Juhl, Annette Ekblond

**Affiliations:** 1 Cardiology Stem Cell Centre, The Heart Centre, Copenhagen University Hospital Rigshospitalet, Henrik Harpestrengs vej 4C, Dept. 9302, 2100 Copenhagen, Denmark; 2 Nordic Bioscience A/S, Herlev Hovedgade 205-207, 2730 Herlev, Denmark; 3 Department of Cardiology, The Heart Centre, Copenhagen University Hospital Rigshospitalet, Blegdamsvej 9, 2100 Copenhagen, Denmark

**Keywords:** Adipose-derived stromal cells, Extracellular matrix, Metalloproteinases, Macromolecular crowding, Fibroblasts, Paracrine and juxtacrine co-cultures

## Abstract

**Background:**

Adipose-derived stromal cells (ASCs) possess a multitude of regenerative capabilities, which include immunomodulation, angiogenesis, and stimulation of extracellular matrix (ECM) remodeling. However, the underlying mechanisms leading to ECM remodeling remain largely elusive and highlight the need for functional in vitro models for mode of action studies. Therefore, the purpose of this study was to develop an in vitro co-culture model to investigate the capabilities of ASCs to modulate fibroblasts and ECM.

**Methods:**

An ECM in vitro model with ASCs and normal human dermal fibroblasts (NHDFs) was established utilizing macromolecular crowding, ascorbic acid, and TGF-β stimulation. Paracrine and juxtacrine co-cultures were created using transwell inserts and cell cultures with direct cell–cell contacts. The cultures were screened using RT^2^ PCR Profiler Arrays; the protein levels of myofibroblast differentiation marker alpha smooth muscle actin (αSMA) and ECM remodeling enzymes were analyzed using western blot on cell lysates; the formation of collagen type I, III, VI, and fibronectin was investigated using ELISA on culture supernatants; and the deposition of collagens was analyzed using immunocytochemistry.

**Results:**

TGF-β stimulation of NHDF monocultures increased the expression of 18 transcripts relevant for ECM formation and remodeling, the protein levels of αSMA and matrix metalloproteinase-2 (MMP-2), the formation of collagen type I, III, VI, and fibronectin, and the deposition of collagen type I and VI and decreased the protein levels of MMP-14. Inclusion of ASCs in the ECM co-culture model increased the formation of collagen type I and III through paracrine mechanisms and the formation of collagen type VI through juxtacrine mechanisms.

**Conclusions:**

The co-culture model provides effective stimulation of NHDF monocultures by TGF-*β* for enhanced formation and deposition of ECM. In the model, ASCs induce changes in ECM by increasing formation of collagen type I, III and VI. The obtained results could guide further investigations of ASCs’ capabilities and underlying mechanisms related to ECM formation and remodeling.

**Supplementary Information:**

The online version contains supplementary material available at 10.1186/s13287-022-02923-y.

## Introduction

Adipose-derived stromal cells (ASCs) are mesenchymal cells that carry multiple regenerative, immunomodulatory, and pro-angiogenic capabilities. This makes them attractive cell therapy candidates for different clinical indications, including treatment of chronic ischemic heart disease and diabetic foot ulcers [1–3]. These diseases may share common underlying features such as dysregulated immune responses and pathological extracellular matrix (ECM) remodeling [4, 5]. The ECM is not just a passive structural component, but a dynamic entity which is constantly reorganized and affects various biological and cellular processes including cell adhesion, migration, proliferation, and differentiation. Hence, formation and remodeling of ECM is a prerequisite for wound healing, angiogenesis and tissue regeneration [6, 7], which might suggest a regenerative mode of action of ASCs involving modulation of ECM [8].

The ECM is produced and remodeled by fibroblasts which secrete ECM components and remodeling enzymes [9], including matrix metalloproteinases (MMPs) that degrade ECM, and tissue inhibitors of MMPs (TIMPs) that reduce MMP activity [10]. Transforming growth factor β1 (TGF-β) plays important roles in ECM remodeling and stimulates myofibroblast differentiation and ECM production [11]. The main constituents of ECM are interlocked in a complex network of proteins and sugars, with collagens being the most abundant protein present [12]. Especially neosynthesis of collagen type I is greatly increased in wound healing and fibrosis, and its expression is correlated with several other ECM components [13].

Interestingly, studies using animal models have shown that ASCs improve wound healing [14, 15] and reduce fibrosis and scarring [4, 16–18]. These abilities have also been demonstrated in clinical trials [3, 19, 20]. This suggests profound effects on ECM. However, knowledge of the mechanisms behind the regenerative effects on ECM remains scarce. Discovery of these will support the development of functional regenerative assays for quality control of ASC therapeutics [21].

The regenerative capabilities of ASCs are primarily believed to be mediated through secretion of paracrine factors, but some effects might be mediated through direct, juxtacrine cell–cell contacts [5, 22–25]. Through these mechanisms, ASCs induce changes in fibroblasts and myofibroblasts, altering their production of ECM, MMPs and TIMPs, and their differentiation status and survival [22, 26, 27]. ASCs themselves also produce ECM, MMPs and TIMPs that contribute to the regenerative process [28, 29].

The need for functional ECM models may be fulfilled by in vitro models; however, standard cell culture methodologies do not reflect the physiologically crowded states that cells experience in vivo [30–33]. The lack of physical cues results in deviating cell function. Fibroblasts cultured in conventional medium may produce all the required precursors for ECM production, yet deposition and crosslinking to form the intricate structural matrices characteristic for ECM is limited [30]. Introducing macromolecular crowding (MMC) augment the generation of ECM through the excluded volume effect, which accelerates enzymatic reactions and assembly of molecules into organized structures, resulting in enhanced ECM deposition in vitro [30–33].

Combining the physical and chemical cues of MMC, ascorbic acid, and TGF-β stimulation with the use of transwell inserts resulted in a model which allowed for investigation of paracrine and juxtacrine cell interactions between ASCs and the fibroblast responder cells of the co-cultures. The model demonstrated that TGF-β stimulation induces multiple changes in the fibroblast monocultures, and that the inclusion of ASCs mediates increased formation of collagens through paracrine and juxtacrine mechanisms.

## Materials and methods

### Cell culture

Normal human dermal fibroblasts (NHDFs, Promocell) were cultured in Dulbecco’s Modified Eagle’s Medium (DMEM) with 10% fetal bovine serum (FBS), and 100 U/ml penicillin and 100 µg/ml streptomycin (P/S), all from Gibco. ASCs were isolated by enzymatic digestion of lipoaspirate from five consenting healthy volunteers as described previously [34], and cultured in Minimal Essential Medium alpha (αMEM, Gibco) with 5% human platelet lysate (Stemulate, Sexton Biotechnologies), and P/S. Information regarding the ASC donors is included in Additional file 1A.

For all experimental conditions, NHDFs and ASCs were seeded as monocultures, paracrine co-cultures, or juxtacrine co-cultures in 24-well plates (µ-Plate, Ibidi) in DMEM with 10% FBS, and P/S. For paracrine co-cultures, 25,000 NHDFs were seeded in bottom wells and 5,000 ASCs were seeded in transwell cell culture inserts with 0.4 µm pores (Nunc). For juxtacrine co-cultures, 25,000 NHDFs and 5,000 ASCs were seeded simultaneously in bottom wells. For controls, 25,000 NHDFs were seeded as monocultures in bottom wells. For ASC monocultures, 5,000 ASCs were seeded in inserts with no cells in the bottom wells. The only exception was for western blot and immunocytochemistry experiments, where 25,000 ASCs were seeded as monocultures in bottom wells. The day after seeding, cells were washed with phosphate buffered saline (PBS^−Ca2+/Mg2+^, Gibco), and MMC was introduced by addition of 37.5 mg/ml Ficoll 70 (Sigma) and 25 mg/ml Ficoll 400 (Sigma) in DMEM with 0.4% FBS, P/S, and 100 uM L-ascorbic acid phosphate magnesium salt n-hydrate ascorbic acid (Wako), with or without 5 ng/ml human recombinant TGF-β1 (Sigma) (day 0), as developed by Chen et al*.* [30]. NHDF monocultures, ASC monocultures, paracrine co-cultures and juxtacrine co-cultures were stimulated with TGF-β. Unstimulated NHDF monocultures were included as a control to demonstrate the responsiveness of the model. Supernatants were harvested at day 3, 6, 10, and 13, and fresh media with ficolls, ascorbic acid, with or without TGF-β were added (100% media change). Supernatants were stored at − 80 °C.

### Flow cytometry

ASCs from five donors were characterized by flow cytometry, according to the ISCT guidelines. Briefly, the cells were thawed, washed in PBS and stained with the viability dye FVS780 (BD) for 10 min at room temperature (RT) in the dark. Cells were washed in fluorescence activated cell sorting (FACS)-buffer containing FACS-PBS (Hospital Pharmacy), 1% ethylenediaminetetraacetic acid (EDTA) (Hospital Pharmacy), supplemented with 10% γ-irradiated and heat-inactivated FBS (Gibco, Life Technologies). Next, the cells were centrifuged at 300 G for 5 min at RT, resuspended in FACS-buffer and stained for 20–30 min. at RT in the dark with the following antibodies in separate tubes: CD73-APC (AD2), CD90-FITC (5E10), CD105-FITC (266), CD45-PE (HI30) and HLA-DR,DP,DQ-BV421 (TÜ39). Finally, the cells were washed in FACS-buffer, centrifuged and resuspended in PBS. The cells were acquired on a FACS Lyric flow cytometer (BD), and data were analyzed using Flow Logic software (Inivai Technologies) based on 10,000 single and live cells. ASCs expressed high levels (> 80%) of CD73, CD90, and CD105, and low levels (< 2%) of CD45 and HLA-DR-DP-DQ (Additional file 1B).

### ELISA

Biomarkers of ECM formation were assessed in supernatants by competitive enzyme-linked immunosorbent assays (ELISAs) developed at Nordic Bioscience A/S, Denmark [35–38]. The PRO-C1 assay detects an internal epitope in the N-terminal pro-peptide of collagen type I, which is released during extracellular processing of pro-collagen to collagen, indicative of collagen type I formation. The PRO-C3 assay detects the released N-terminal pro-peptide of collagen type III, indicative of collagen type III formation. The PRO-C6 assay detects the C-terminal of released C5 domain of collagen type VI α3 chain (also known as endotrophin), indicative of collagen type VI formation. The FBN-C assay detects the C-terminal of fibronectin, indicative of fibronectin formation [31]. 96-well streptavidin coated plates were coated with assay-specific coater, incubated for 30 min., and washed. Standard curve, controls and samples were loaded in duplicates, followed by addition of HRP-labelled antibody, and the plates were incubated for 1–20 h., depending on the assay. Next, plates were washed and incubated with Tetramethylbenzinidine (TMB ONE, Kem-En-Tec) for 15 min. The reactions were stopped using 1% HCl solution. Plates were measured at 450 nm with 650 nm as a reference (Molecular Devices, SpectraMax M). A standard curve was plotted using a 4-parametric mathematical fit model.

### Immunocytochemistry

Cells were fixed in 4% paraformaldehyde in PBS (Hospital pharmacy, Copenhagen University Hospital) for 15 min. at RT, permeabilized in 0.3% TritonX-100 in PBS (Sigma) for 15 min. at 4 °C and blocked in 1% (w/v) bovine serum albumin in PBS (Sigma), followed by incubation with the following primary antibodies for 1 h. at RT: Mouse monoclonal anti-human collagen type I, Rabbit polyclonal anti-human collagen type III, and Rabbit monoclonal anti-human collagen type VI (SD83-03) (all from Thermo Fisher). Next, cells were washed and incubated with secondary antibodies for 1 h. at RT in the dark: Goat anti-mouse IgG (H + L) cross-adsorbed secondary antibody, alexa fluor 488, or Goat anti-rabbit IgG (H + L) highly cross-adsorbed secondary antibody, alexa fluor 546 (both from Thermo Fisher). Lastly, cells were stained with DAPI (Thermo Fisher) for 5 min. Details regarding the antibodies are included in the Additional file 2A. In order to quantify cell numbers, additional monocultures of unstimulated and TGF-β stimulated NHDFs were cultured in parallel, and fixed at day 3, 6, 10, and 13, followed by DAPI staining.

### Microscopy and image analysis

Cells were imaged using an inverted microscope (Axio Observer 7, Zeiss) fitted with scanning stage, microLED illumination for transmitted light, and HXP 120 V fluorescence light source. Using a Plan-Apochromat 20x/0.8 Ph2 objective and Axiocam 506 mono camera, stitched images of 7 × 7 tiles were obtained covering 26 mm^2^ of each well. Separate channels were acquired for alexa fluor 488, alexa fluor 546, and DAPI. Exposure times were fixed between samples, allowing for comparison of intensities.

For quantification of deposited ECM, the mean gray values of acquired images were determined in Fiji version 1.49 m [39]. For quantification of cell numbers, acquired DAPI images were analyzed in Fiji, by employing a macro for automatic background subtraction, Otzu threshold, watershed, and particle analysis.

### Western blot

Cells were lysed in Tris-buffered saline with 1% Triton X-100 and protease inhibitor cocktail (Thermo Fisher), and protein concentrations were measured with DC protein Assay (Bio-Rad) on FLUOstar Omega plate reader (BMG Labtech). Western blot was performed by SDS-PAGE on XCell SureLock mini using NuPAGE 4–12% Bis–Tris Plus gels and NuPAGE MES SDS running buffer. Proteins were transferred onto PDVF membranes using iBlot 2 Gel Transfer Device (all from Thermo Fisher). Blots were blocked in 5% (w/v) non-fat dry milk for 1 h. at RT, and incubated with the following primary antibodies overnight at 4 °C: Mouse monoclonal anti-human alpha smooth muscle actin (1A4), Rabbit polyclonal anti-human MMP-14, Mouse monoclonal anti-human MMP-2 (CA-4001 (CA719E3C)), Mouse monoclonal anti-human TIMP-1 (102D1), Mouse monoclonal anti-human TIMP-2 (3A4), Rabbit polyclonal anti-human beta tubulin, Mouse monoclonal anti-human GAPDH (GA1R), Mouse monoclonal anti-human beta actin (BA3R) (all from Thermo Fisher), Mouse monoclonal anti-human vinculin (VIN-11–5) (Sigma-Aldrich). Next, blots were washed and incubated with the following secondary antibodies for 1 h, at RT: Goat anti-mouse IgG (H + L) cross-adsorbed secondary antibody, HRP or Goat anti-rabbit IgG (H + L) cross-adsorbed secondary antibody, HRP (both from Thermo Fisher). Protein bands were detected using SuperSignal West Pico PLUS or SuperSignal West Femto Chemiluminescent Substrates (Thermo Fisher) and developed using a ChemiDoc XRS + molecular Imager (Bio-Rad). Antibodies, antibody concentrations, amounts of loaded protein, and the used detection substrates and exposure times are included in Additional file 2B. Full-length blots are included in Additional file 3.

Densitometric quantification of protein bands was performed in Fiji and compared to the mean of loading controls as a target/control ratio.

### First strand synthesis and RT^2^ profiler PCR array

Total RNA was extracted with RNeasy Micro Kit (Qiagen). RNA purity and concentration were determined with Nanodrop 1000 Spectrophotometer (Thermo Fisher) by absorbance ratios A260/A280 and A260/A230, and RIN values using 2100 Bioanalyzer instrument (Agilent) and 2100 Expert Software (Agilent). cDNA synthesis was performed with RT^2^ First Strand kit (Qiagen) on Veriti 96-well fast thermal cycler (Applied Biosystems) at 42 °C for 15 min., and subsequently at 95 °C for 5 min.

Forty target genes relevant for ECM formation and remodeling were chosen for custom made RT^2^ Profiler PCR arrays (Qiagen). The investigated genes are included in the Additional file 4C. cDNA, RT^2^ SYBR Green Mastermix (Qiagen), and RNase-free water (Fisher BioReagents) were mixed according to manufacturer’s instructions, reactions were run on a PCR plate reader (Bio-Rad, CFX Connect™), and data were analyzed using CFX Maestro Software (Bio-Rad). A two-step amplification cycle was applied, with initial denaturation at 95 °C for 10 min., and subsequently 40 cycles of denaturation at 95 °C for 15 s. and annealing and elongation at 60 °C for one min. Beta actin, RPLP0 and GAPDH were included as potential reference genes due to their stability in fibroblasts and bone marrow-derived mesenchymal stromal cells at crowded conditions [33, 40, 41]. Beta actin and GAPDH were chosen due to their stability between the experimental groups (combined stability value = 0.144, using Normfinder version 0.953) [42].

### Statistics

Normality was assessed with Shapiro–Wilk Tests of Normality. Equality of variances was assessed with Levene’s Test. ELISA data and cell count data from NHDF monocultures were analyzed using repeated measures ANOVA. Quantified immunocytochemistry data and quantified western blot data were analyzed using independent samples t-test. Above tests were performed using IBM SPSS Statistics version 25. Comparison of ELISA data from co-cultures with the sum of data from NHDF monocultures and ASC monocultures was performed using mixed models in SAS version 9.4. RT^2^ Profiler PCR Array data were analyzed using GeneGlobe (Qiagen, assessed August 2021), in which Cq values of the genes of interest were normalized to the geometric mean of the reference genes. Differences in expression levels were calculated using independent samples t-test of 2^−ΔCq^ values between the experimental groups. Graphs and plots were created using SPSS and GeneGlobe. Data are presented as mean ± standard error. A *p*-value < 0.05 was considered statistically significant.

## Results

### Screening for differentially regulated genes using RT^2^ profiler PCR arrays

**Fig. 1:**
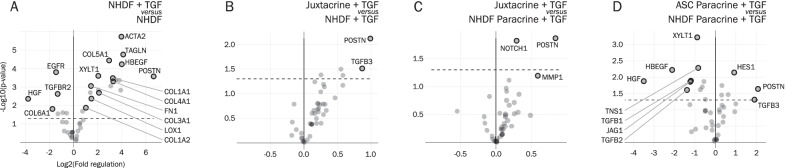
Results from custom RT^2^ PCR Profiler Arrays, depicting volcano plots of differentially regulated genes between the different experimental groups. Cells were harvested at day 10. **A** NHDF + TGF-β vs NHDF. **B** Juxtacrine + TGF-β vs NHDF + TGF-β. **C** Juxtacrine + TGF-β vs NHDF Paracrine + TGF-β. **D** ASC Paracrine + TGF-β vs NHDF Paracrine + TGF-β. Based on experiments with three ASC donors (paracrine co-cultures, juxtacrine co-cultures, and ASC monocultures), with one replicate per ASC donor, and three replicates for NHDF monocultures with or without TGF-β. Significance level: *p < 0.05

Screening for differentially regulated genes using custom made RT^2^ Profiler PCR Arrays revealed that the transcripts of many genes were upregulated in TGF-β stimulated NHDF monocultures, relative to unstimulated NHDF monocultures (Fig. 1A). These included myofibroblast activation and differentiation marker alpha smooth muscle actin (αSMA) (encoded by ACTA2), cytoskeletal associated protein transgelin (TAGLN), heparin-binding EGF-like growth factor (HBEGF), matricellular protein periostin (POSTN), secreted ECM including collagen type I (Col1A1 and Col1A2), III (Col3A1), IV (Col4A1), V (Col5A1), and fibronectin (FN1), ECM producing and crosslinking enzymes xylosyltransferase I (XYLT1) and lysyl oxidase (LOX1), and others. Fewer genes were downregulated, including hepatocyte growth factor (HGF), epidermal growth factor receptor (EGFR), TGF-β receptor II (TGFBR2), and collagen type VI (Col6A1).

Surprisingly, there was no difference in expression in NHDFs from paracrine co-cultures (NHDF Paracrine + TGF-β), relative to TGF-β stimulated NHDF monocultures (Additional file 4, left). In juxtacrine co-cultures, the expression of POSTN was increased, relative to TGF-β stimulated NHDF monocultures (Fig. 1B). The same trend on POSTN was observed in juxtacrine co-cultures, relative to NHDFs from paracrine co-cultures (Fig. 1C). However, a direct comparison between juxtacrine co-cultures and NHDFs cannot be made, due to the presence of ASCs in the juxtacrine co-cultures. In fact, the increase in POSTN in juxtacrine co-cultures was likely due to a direct contribution from ASCs. ASCs from paracrine co-cultures expressed higher levels of POSTN, relative to NHDFs from paracrine co-cultures (Fig. 1D), while there was no difference in POSTN in juxtacrine co-cultures, relative to ASCs from paracrine co-cultures (Additional file 4, right).

Surprisingly, the transcript of HGF, a pro-angiogenic and anti-fibrotic factor present in the ASC secretome, was lower in ASCs from paracrine co-cultures, relative to NHDFs from paracrine co-cultures (Fig. 1D).

### Effects of ASCs on myofibroblast differentiation and ECM remodeling enzymes

**Fig. 2:**
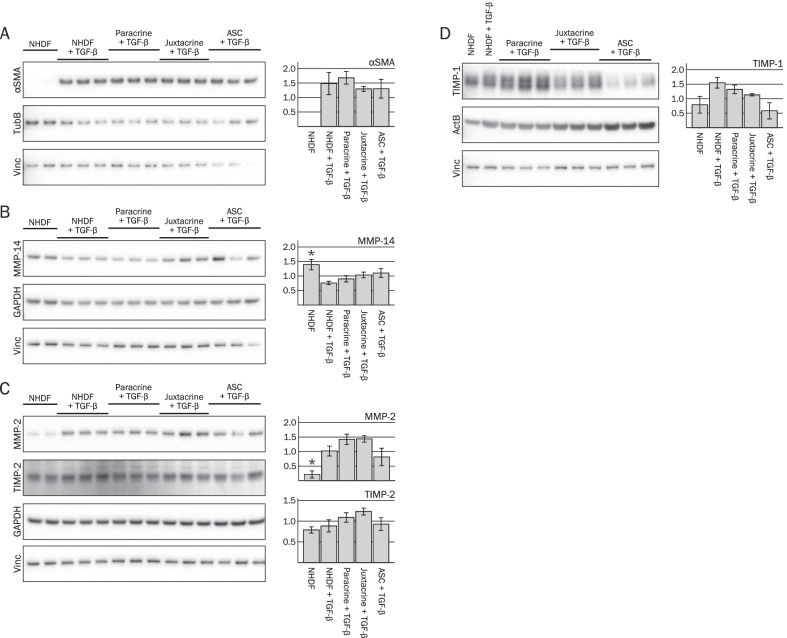
Effects of ASCs on protein levels of αSMA and ECM remodeling enzymes, investigated by western blot at day 10. The quantified data are based on lysates from three independent experiments. The same three ASC donors were included in experiment 1 and 2. In experiment 3, two additional ASCs donors were used (*n* = 5). **A**–**D** Representative images of blots are shown. For instance, the images found in **A** represent a single blot (Blot A), which has been cut into three parts prior to incubation with anti-αSMA, anti-TubB, and anti-Vinc, respectively. The same is the case for the blots shown in **B**–**D**. Each replicate lane in the shown blots for NHDF Paracrine + TGF-β, Juxtacrine + TGF-β, and ASC + TGF-β represent lysates from co-cultures with three different ASC donors, respectively. The quantified bands of target proteins were related to the mean of the loading controls. Protein lysates from each experiment were loaded onto four gels/blots (designated A-D) as illustrated. Significance level: *p < 0.05, compared to NHDF + TGF-β. Abbreviations: TubB, Beta tubulin; Vinc, Vinculin; GAPDH, glyceraldehyde 3-phosphate dehydrogenase; ActB, beta actin

Protein levels of the myofibroblast activation and differentiation marker αSMA and ECM remodeling enzymes were investigated using western blot. Stimulation with TGF-β increased the protein levels of αSMA in NHDF monocultures, although not significantly (*p* = 0.061) (Fig. 2A). Addition of ASCs did not affect αSMA levels in NHDFs from paracrine co-cultures (NHDF paracrine + TGF-β), or in the juxtacrine co-cultures (Juxtacrine + TGF), compared to the TGF-β stimulated NHDF monocultures (*p* = 0.643 and *p* = 0.676, respectively).

TGF-β stimulation decreased the levels of MMP-14 in NHDF monocultures (*p* = 0.028) (Fig. 2B). Addition of ASCs in inserts did not affect MMP-14 in NHDFs from paracrine co-cultures (NHDF Paracrine + TGF-β), while MMP-14 levels tended to be increased in juxtacrine co-cultures (*p* = 0.384 and *p* = 0.080, respectively). However, this trend might be a result of MMP-14 expression in ASCs present in the juxtacrine co-cultures, as the MMP-14 levels in ASCs (ASC + TGF-β) appear higher than in NHDFs (NHDF + TGF-β).

TGF-β stimulation increased the levels of MMP-2 in NHDF monocultures (*p* = 0.018) (Fig. 2C). While addition of ASCs in inserts did not affect the MMP-2 levels in NHDFs from paracrine co-cultures, the MMP-2 levels in juxtacrine co-cultures tended to be increased (*p* = 0.187 and *p* = 0.086, respectively).

TGF-β stimulation tended to increase TIMP-1 levels but did not affect TIMP-2 levels in NHDF monocultures (*p* = 0.092 and *p* = 0.582, respectively) (Fig. 2C, D)levels of TIMP-1 and TIMP-2 were unchanged in NHDFs from paracrine co-cultures, compared to the TGF-β stimulated NHDF monocultures (*p* = 0.369 and *p* = 0.315, respectively). Lastly, there was no effect on TIMP-1 in juxtacrine co-cultures, while TIMP-2 tended to be increased (*p* = 0.146 and *p* = 0.078, respectively).

### Inclusion of ASCs increases the formation of collagens through paracrine and juxtacrine mechanisms

**Fig. 3:**
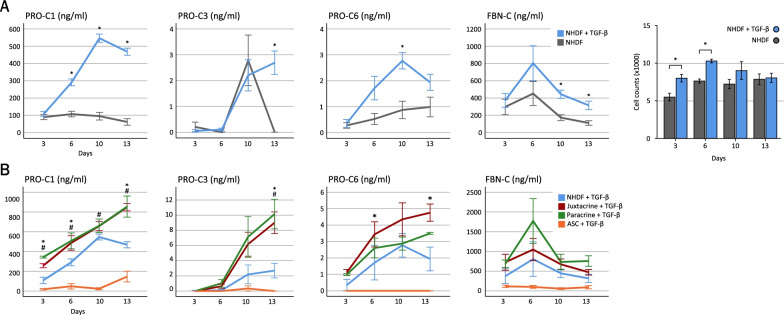
Effects of ASCs on ECM formation, measured in supernatants by ELISA at day 3, 6, 10, and 13. **A**, left) ECM formation by TGF-β stimulated NHDF monocultures, compared to unstimulated NHDF monocultures. Based on data from two experiments, with a minimum of four replicate wells per experiment per condition. Significance level: *p < 0.05. A, right) Quantification of cell numbers in TGF-β stimulated and unstimulated NHDF monocultures, based on DAPI staining. Eight replicate wells were stained per condition per day. Data for day 3, 6, and 13 are based on one experiment. Data for day 10 are based on two experiments. Significance level: *p < 0.05. **B**) ECM formation in co-cultures, compared to the sum of the levels in NHDF monocultures and ASC monocultures. Based on data from two experiments with a minimum of four replicate wells per experiment per condition, three ASC donors were included in experiment 1 and measurements were performed at day 3, 6, 10, and 13. In experiment 2, two ASC donors were included, and measurements were performed at day 6 and 10 (*n* = 5 for day 6 and 10, *n* = 3 for day 3 and 13). Significance level: juxtacrine co-cultures vs. the sum of monocultures: *p < 0.05. Paracrine co-cultures vs. the sum of monocultures: ^#^*p* < 0.05

The formation of ECM was measured by quantifying soluble neoepitopes indicative of collagen and fibronectin formation in supernatants (Fig. 3). TGF-β stimulation of NHDF monocultures increased the levels of collagen type I (PRO-C1) at day 6, 10, and 13 (*p* < 0.001), the levels of collagen type III (PRO-C3) at day 13 (*p* < 0.001), and the levels of collagen type VI (PRO-C6) at day 10 (*p* = 0.001) (Fig. 3A). The levels of fibronectin (FBN-C) were also increased by TGF-β stimulation at day 10 and 13 (*p* = 0.001 and *p* = 0.021). Simultaneously, parallel cultures of macromolecularly crowded NHDF monocultures were fixed, stained with DAPI, and subjected to image analysis to quantify cell numbers. This showed that cell numbers were higher in TGF-β stimulated cultures at day 3 and 6 (*p* = 0.024 and *p* = 0.027, respectively), while there was no significant difference at day 10 and 13 (*p* = 0.087 and *p* = 0.388, respectively). Further, the large differences in ECM formation between TGF-β stimulated and unstimulated cultures were not reflected in the relatively small differences in cell numbers. Thus, while proliferation may contribute to the increased ECM formation it is most likely primarily ascribed to increased production.

In order to investigate the effects of ASCs on ECM formation, these were added and the levels of biomarkers in supernatants of TGF-β stimulated co-cultures were compared to the combined levels in TGF-β stimulated NHDF monocultures and TGF-β stimulated ASC monocultures (Fig. 3B). This showed that the PRO-C1 levels were increased in juxtacrine and paracrine co-cultures, compared to the combined levels in monocultures (juxtacrine; day 3, *p* < 0.001; day 6, *p* = 0.030; day 13, *p* = 0.012), (paracrine; day 3, *p* < 0.001; day 6, *p* = 0.030; day 10, *p* = 0.045; day 13, *p* = 0.010). The levels of PRO-C3 tended to be increased in paracrine co-cultures at day 10 and were increased in juxtacrine and paracrine co-cultures at day 13 (paracrine; day 10 *p* = 0.096; day 13, *p* < 0.001), (juxtacrine; day 10, *p* = 0.562; day 13, *p* < 0.001). Interestingly, PRO-C6 levels were increased in juxtacrine co-cultures, compared to monocultures (day 6, *p* < 0.001; day 13, *p* < 0.001), while PRO-C6 levels in paracrine co-cultures were the same as in monocultures. Finally, there were no differences in FBN-C levels between co-cultures and monocultures.

### Effects of ASCs on ECM deposition

**Fig. 4:**
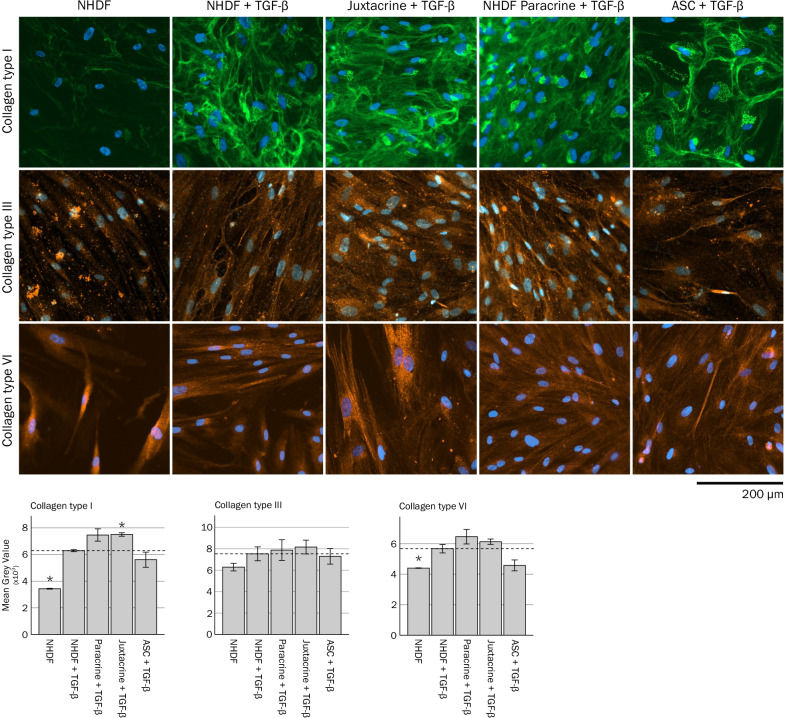
Effects of ASCs on deposition of collagen type I, III, and VI, investigated by immunocytochemistry at day 10. Data for unstimulated and TGF-β stimulated NHDF monocultures are based on nine replicate wells per condition. Data for co-cultures and for ASC monocultures are based on experiments with four ASC donors with three replicate wells per donor per condition. Significance level: *p < 0.05, compared to NHDF + TGF-β

The presence of collagen type I, III and VI protein was investigated using immunocytochemistry (Fig. 4 and Additional file 5). Staining of collagen type I in cultures revealed fibrillar-like structures extracellularly, and some staining was also evident intracellularly, with a more diffuse granular appearance located close to the cell nuclei (Fig. 4 and Additional file 6). TGF-β stimulation increased the presence of extracellular collagen type I fibrils and intracellular collagen type I in NHDF monocultures, compared to unstimulated NHDF monocultures (as evident by an increase in mean gray values, *p* < 0.001). Addition of ASCs in inserts tended to increase the presence of collagen type I in the bottom wells (NHDF Paracrine + TGF compared to NHDF + TGF) (*p* = 0.083). The presence of collagen type I was increased in juxtacrine co-cultures, compared to TGF-β stimulated NHDF monocultures (*p* = 0.001).

Collagen type III was present intracellularly, and/or closely located to the cell membranes extracellularly in the cultures. Visually, TGF-β stimulation appeared to increase collagen type III in NHDF monocultures, compared to unstimulated NHDF monocultures, but the effect was not significant (*p* = 0.167). Addition of ASCs did not affect the levels of collagen type III in paracrine or juxtacrine co-cultures, compared to TGF-β stimulated NHDF monocultures (*p* = 0.796 and *p* = 0.530, respectively).

Like collagen type III, collagen type VI was also located intracellularly, and/or closely to the cell membranes extracellularly. TGF-β stimulation increased the levels of collagen type VI in NHDF monocultures, compared to unstimulated NHDF monocultures (*p* = 0.010). However, addition of ASCs did not affect the collagen type VI levels in paracrine or juxtacrine co-cultures, compared to TGF-β stimulated NHDF monocultures (*p* = 0.216 and *p* = 0.202, respectively).

## Discussion

Despite advances in conventional medical treatments, there still is a need for novel regenerative strategies for improvement of wound healing, reduction of fibrosis, and stimulation of tissue regeneration in patients. ASC therapy represents one of these strategies, in which ASCs are applied alone or in combination with exogenic growth factors or scaffolds [43]. It is becoming increasingly apparent that the ECM is crucial for cell behavior and tissue function and affects various biological processes including wound healing, angiogenesis, fibrosis, and tissue regeneration [6, 7]. The capability of ASCs to promote wound healing and reduce fibrosis thus suggests mode of actions involving modulation of ECM [8, 14]. To investigate this capability, an in vitro ECM co-culture model of ASCs and NHDFs was developed. The model allows investigation of paracrine and juxtacrine cell interactions, which revealed effects on formation of three distinct ECM molecules. The ECM co-culture model has several physical and chemical cues present, including MMC, ascorbic acid, and provides effective induction by TGF-β. The advantages of MMC include enhanced conversion of pro-collagen to collagen by C-proteinases, and increased crosslinking of collagen fibers by LOX, which results in increased collagen formation and deposition [30]. The responsiveness of the model was further demonstrated by the multiple changes at protein and RNA levels, and multiple changes in ECM in the NHDF monocultures induced by TGF-β.

Inclusion of ASCs in the model increased the formation of collagen type I in both paracrine and juxtacrine co-cultures, as measured by increased levels of PRO-C1 in the supernatants. Immunocytochemical analysis further revealed that the levels of collagen type I located intracellularly, or extracellularly as fibrils, tended to be increased in paracrine co-cultures and was increased in juxtacrine co-cultures. As paracrine interactions take place in both paracrine and juxtacrine co-cultures, while ASCs and NHDFs are physically separated in paracrine co-cultures, these results indicate that the increase in collagen type I formation primarily is mediated through paracrine capabilities of ASCs.

Among the ~ 20 subtypes of collagen, collagen type I is the most abundant, and the primary structural component of ECM, and it is part of both physiological and pathological ECM [44]. Inclusion of ASCs also increased the levels of PRO-C3 in the supernatants at day 13, indicative of increased formation of collagen type III, through paracrine mechanisms. Immunocytochemical analysis did not reveal an increase in deposited collagen type III in co-cultures at day 10. However, it might have been possible to detect increased collagen type III deposition at day 13, as the ELISA data revealed increasing formation of collagens with increased culture duration. This remains to be determined. Collagen type III is involved in regulation of ECM assembly [45].

PRO-C6 was increased in the supernatants of juxtacrine co-cultures at day 6 and 13, indicative of increased collagen type VI formation induced via juxtacrine mechanisms. An increase in intracellular and extracellular collagen type VI could not be detected by immunocytochemical staining in juxtacrine co-cultures at day 10, despite the potential contribution from ASCs. The effects on deposition at day 13 remains to be determined.

Collagen type VI regulates ECM assembly and organization and interacts with a variety of ECM including collagen type I, II, and IV, fibronectin, glycosaminoglycans and proteoglycans. Collagen type VI has been suggested to regulate the direction of migration in tendon fibroblasts [45, 46], and to accelerate the migration of lung epithelial cells [47] and macrophages [48]. Thus, the increased formation of collagen type VI induced by ASCs might suggest a mechanism for induction of cell migration. ASCs are known to possess immunomodulatory capabilities and induce polarization of reparative M2 macrophages [49]. Interestingly, the polarization of M2 macrophages is promoted by collagen type VI [48].

The increased formation of collagens in the co-cultures was not accompanied by increased expression of the corresponding transcripts. This might suggest upregulation at the protein level, rather than at the RNA level. The increased formation could also be a result of increased NHDF proliferation induced by ASCs. This needs to be determined in future studies. However, the different mechanisms of induction (paracrine for collagen type I and III vs. juxtacrine for collagen type VI), and the lack of effect on fibronectin, might suggest that induction of proliferation is not the sole mechanism behind these capabilities. ASCs have been shown to promote wound healing in animal models and clinical trials [3, 14, 15, 19]. For instance, ASCs were shown to accelerate granulation tissue formation in a murine full-thickness excisional wound model [15]. One of the mechanisms behind this capability presumably involves stimulation of fibroblast migration, proliferation and ECM production, which is characteristic for the proliferation phase of wound healing [3, 8, 50]. Thus, the ECM co-culture model of NHDFs and ASCs might be utilized to study mechanisms of ASCs during this particular phase of the wound healing response.

The observed juxtacrine capabilities of ASCs suggest that conditioned medium cannot fully replace ASC therapy. Once the ASCs are injected, however, the cell retention might be low, which decreases the probability of forming juxtacrine cell–cell contacts with resident cells of the tissue. Combining ASC therapy with a scaffold might increase cell retention and survival, thereby enhancing cell interactions and formation of collagens, for enhanced regenerative effects [51].

POSTN was increased in juxtacrine co-cultures, relative to TGF-β stimulated NHDF monocultures, even though this might have been caused by a contribution from ASCs present in juxtacrine co-cultures. POSTN is essential for wound healing. Following an increased expression during the proliferative phase, the levels gradually reduce during the terminal remodeling phase; however, when the levels fail to decline, fibrosis entails [52]. Besides the role in wound healing, the production of POSTN by ASCs has been linked to other functions, e.g., osteogenic differentiation potential [53] and progression of B-cell acute leukemia through modulation of CCL2 signaling [54].

TGF-β stimulation upregulated many transcripts in NHDF monocultures and downregulated the anti-fibrotic and pro-angiogenic factor HGF. It was surprising, though, that HGF was lower in ASCs from paracrine co-cultures, relative to NHDFs from paracrine cultures. This might indicate that ASC-secreted HGF does not contribute in this model. Still, the secreted levels of HGF need to be investigated. In addition, the RT^2^ Profiler PCR Array is a screening tool, and the results must be validated in future studies.

The protein levels of αSMA were not affected in paracrine or juxtacrine co-cultures, which suggests that there was no effect of ASCs on myofibroblast differentiation. This is in line with a study using ASC conditioned medium and TGF-β stimulated cardiac fibroblasts at non-crowded conditions [55]. Likewise, the protein levels of MMP-14, MMP-2, TIMP-1 and TIMP-2 were not affected in the co-cultures. Still, the effects on secreted levels of these ECM remodeling enzymes remain to be determined.

When comparing juxtacrine co-cultures to NHDF monocultures, it is important to keep in mind that juxtacrine co-cultures contain both NHDFs and ASCs, and therefore, a direct comparison cannot be made. However, as NHDFs were added in excess in juxtacrine co-cultures (25,000 NHDFs compared to 5,000 ASCs), the contribution from ASCs might be relatively negligent. Thus, for western blot results, the detected protein bands for juxtacrine co-cultures would not reflect a direct 1:1 contribution from the NHDF monocultures and ASCs monocultures, respectively.

The presented co-culture model provides several functional outputs for the study of paracrine and juxtacrine mediated effects of ASCs on NHDFs and ECM.

## Conclusion

The co-culture model applied in this study can efficiently be induced to produce ECM by TGF-β stimulation, causing a plethora of effects on NHDFs besides the ECM itself, and it enables investigation of paracrine and juxtacrine capabilities of ASCs. Using this model, we observed that ASCs increased formation of collagen type I and III through paracrine mechanisms, and formation of collagen type VI through juxtacrine mechanisms. The established model and obtained results could guide further investigations of ASC-mediated mechanisms related to ECM formation and remodeling in the future.

## Supplementary Information

**Additional file1.** Additional files.

## Data Availability

The data sets obtained and analyzed in this study are available from the corresponding author upon request.

## Abbreviations

ASC: Adipose-derived stromal cell
αSMA: Alpha smooth muscle actin
DMEM: Dulbecco’s modified eagle’s medium
ECM: Extracellular matrix
FBS: Fetal bovine serum
HGF: Hepatocyte growth factor
LOX: Lysyl oxidase
MMC: Macromolecular crowding
MMP: Matrix metalloproteinase
NHDF: Normal human dermal fibroblast
POSTN: Periostin
P/S: 100 U/ml penicillin and 100 µg/ml streptomycin
RT: Room temperature
TGF-β: Transforming growth factor β1
TIMP: Tissue inhibitors of MMP
