# Supplementary material for: Adipose-derived stromal cells increase the formation of collagens through paracrine and juxtacrine mechanisms in a fibroblast co-culture model utilizing macromolecular crowding
Source: Stem Cell Res Ther. 2022 Jun 11;13:250. doi: 10.1186/s13287-022-02923-y (PMC9188050; doi:10.1186/s13287-022-02923-y)
Supplement: Supplementary file 1 — Additional file1. Additional files. [file 13287_2022_2923_MOESM1_ESM.docx]

# **Adipose-derived stromal cells increase the formation of collagens through paracrine and juxtacrine mechanisms in a fibroblast co-culture model utilizing macromolecular crowding**

Rebekka Harary Søndergaard^1*^, Lisbeth Drozd Højgaard^1^, Alexander Lynge Reese-Petersen^2^, Cecilie Hoeeg^1^, Anders Bruun Mathiasen^3^, Mandana Haack-Sørensen^1^, Bjarke Follin^1^, Federica Genovese^2^, Jens Kastrup^1^, Morten Juhl^1^, Annette Ekblond^1^

**Additional files**

**Figure legends**

**Additional file 1**

A) Information regarding the five ASC donors used in the study. B) Expression of ISCT surface markers on ASCs from five donors.

**Additional file 2**

A) Antibodies for immunocytochemistry. B) Antibodies for western blot. The primary antibodies used for blots A-D respectively are indicated, along with antibody concentrations, amounts of loaded protein, detection substrates and exposure times. C) Genes investigated in the RT^2^ PCR Profiler Arrays.

**Additional file 3**

Full-length blots.

**Additional file 4**

Additional results from the custom RT^2^ PCR Profiler Arrays.

**Additional file 5**

Additional immunocytochemistry images, showing phase contrast and DAPI.

**Additional file 6**

Immunocytochemistry images for comparison of intracellular and extracellular collagen type I and VI. Arrows indicate dense, fibrillar structures typical for ECM. Crude regions containing a single cell are highlighted in white. It should be noted that these does not encompass the entire cell but are devoid of other overlapping cells. Nuclei are mapped in red, and intracellular collagens in green. In order of appearance, the panels depict collagen and nuclei merged, nuclei, phase contrast, and collagen.

**Additional file 1**

**A**

| Number of donors | 5 |
| --- | --- |
| Donor age (years) | 35.6 ± 5.7 (23-55) |
| Sex | 1 male, 4 females |
| Passage | 3-4 |

**B**

|  | % positive |
| --- | --- |
| CD73 | 99.4 ± 0.2 |
| CD90 | 98.4 ± 1.3 |
| CD105 | 87.7 ± 3.1 |
| CD45 | 0.39 ± 0.1 |
| HLA-DR, DP, DQ | 0.16 ± 0.1 |

**Additional file 2**

**A**

| **Primary antibody** | **Reactivity** | **Host** | **Concentration** | **Purchased from** |
| --- | --- | --- | --- | --- |
| Monoclonal anti-Collagen type I | Human | Mouse | 1:2000 dilution of stock (stock:  5 mg/mL) | Thermo Fisher |
| Polyclonal anti-Collagen type III | Human | Rabbit | 1:300 dilution of stock (stock: 1 mg/ml) | Thermo Fisher |
| Monoclonal anti-Collagen type VI Clone SD83-03 | Human | Rabbit | 1:300 dilution of stock (stock: 1 mg/mL) | Thermo Fisher |

| **Secondary antibody** | **Reactivity** | **Host** | **Purchased from** |
| --- | --- | --- | --- |
| Goat anti-mouse IgG (H+L) cross-adsorbed secondary antibody, alexa fluor 488 | Mouse | Goat | Thermo Fisher |
| Goat anti-rabbit IgG (H+L) highly cross-adsorbed secondary antibody, alexa fluor 546 | Rabbit | Goat | Thermo Fisher |

**B**

| **Blot A,**  **Target** | **Reactivity** | **Host** | **Concentration** | **Loaded protein (µg)** | **Antibody purchased from** | **Exposure time (sec.)** | **Detection substrate** |
| --- | --- | --- | --- | --- | --- | --- | --- |
| Monoclonal anti-Alpha smooth muscle actin  Clone 1A4 | Human | Mouse | 1:1000 | 1 | Thermo Fisher | 2.0 | Pico |
| Polyclonal anti-Beta tubulin | Human | Rabbit | 1:2000 | 1 | Thermo Fisher | 34.8 | Pico |
| Monoclonal anti-Vinculin Clone VIN-11-5 | Human | Mouse | 1:1000 | 1 | Sigma-Aldrich | 74.2 | Pico |

| **Blot B, Target** | **Reactivity** | **Host** | **Concentration** | **Loaded protein (µg)** | **Antibody purchased from** | **Exposure time (sec.)** | **Detection substrate** |
| --- | --- | --- | --- | --- | --- | --- | --- |
| Polyclonal anti-MMP-14 | Human | Rabbit | 1:1000 | 16 | Thermo Fisher | 8.0 | Pico |
| Monoclonal anti-GAPDH Clone GA1R | Human | Mouse | 1:5000 | 16 | Thermo Fisher | 2.0 | Pico |
| Monoclonal anti-Vinculin Clone VIN-11-5 | Human | Mouse | 1:1000 | 16 | Sigma-Aldrich | 5.0 | Pico |

| **Blot C, Target** | **Reactivity** | **Host** | **Concentration** | **Loaded protein (µg)** | **Antibody purchased from** | **Exposure time (sec.)** | **Detection substrate** |
| --- | --- | --- | --- | --- | --- | --- | --- |
| Monoclonal anti-MMP-2 Clone CA-4001 (CA719E3C) | Human | Mouse | 5 µg/ml | 25 | Thermo Fisher | 127.0 | Pico |
| Monoclonal anti-TIMP-2 Clone 3A4 | Human | Mouse | 3 µg/ml | 25 | Thermo Fisher | 16.7 | Femto |
| Monoclonal anti-GAPDH Clone GA1R | Human | Mouse | 1:5000 | 25 | Thermo Fisher | 2.0 | Pico |
| Monoclonal anti-Vinculin Clone VIN-11-5 | Human | Mouse | 1:1000 | 25 | Sigma-Aldrich | 3.0 | Pico |

| **Blot D, Target** | **Reactivity** | **Host** | **Concentration** | **Loaded protein (µg)** | **Antibody purchased from** | **Exposure time (sec.)** | **Detection substrate** |
| --- | --- | --- | --- | --- | --- | --- | --- |
| Monoclonal anti-TIMP-1 Clone 102D1 | Human | Mouse | 1.5 µg/ml | 16 | Thermo Fisher | 298.7 | Femto |
| Monoclonal anti-Beta Actin Clone BA3R | Human | Mouse | 1:1000 | 16 | Thermo Fisher | 27.0 | Pico |
| Monoclonal anti-Vinculin Clone VIN-11-5 | Human | Mouse | 1:1000 | 16 | Sigma-Aldrich | 94.5 | Pico |

| **Secondary antibodies (Blots A-D)** | **Reactivity** | **Host** | **Purchased from** |
| --- | --- | --- | --- |
| Goat anti-mouse IgG (H+L) cross-adsorbed secondary antibody, HRP | Mouse | Goat | Thermo Fisher |
| Goat anti-rabbit IgG (H+L) cross-adsorbed secondary antibody, HRP | Rabbit | Goat | Thermo Fisher |

**C**

| **Target** | **Full name** | **NM template sequence** |
| --- | --- | --- |
| TGFB1 | Transforming growth factor, beta 1 | NM_000660 |
| TGFBR1 | Transforming growth factor, beta receptor 1 | NM_004612 |
| TGFBR2 | Transforming growth factor, beta receptor II (70/80kDa) | NM_003242 |
| PDGFA | Platelet-derived growth factor alpha polypeptide | NM_002607 |
| PDGFB | Platelet-derived growth factor beta polypeptide | NM_002608 |
| HGF | Hepatocyte growth factor (hepapoietin A; scatter factor) | NM_000601 |
| CTGF | Connective tissue growth factor | NM_001901 |
| CYR61 | Cyclin-dependent kinase inhibitor 2C (p18, inhibits CDK4) | NM_078626 |
| WISP2/CCN5 | WNT1 inducible signaling pathway protein 2 | NM_003881 |
| TGFB2 | Transforming growth factor, beta 2 | NM_003238 |
| TGFB3 | Transforming growth factor, beta 3 | NM_003239 |
| MMP1 | Matrix metallopeptidase 1 (interstitial collagenase) | NM_002421 |
| COL1A1 | Collagen, type I, alpha 1 | NM_000088 |
| COL1A2 | Collagen, type I, alpha 2 | NM_000089 |
| COL6A1 | Collagen, type VI, alpha 1 | NM_001848 |
| COL3A1 | Collagen, type III, alpha 1 | NM_000090 |
| COL5A1 | Collagen, type V, alpha 1 | NM_000093 |
| COL4A1 | Collagen, type IV, alpha 1 | NM_001845 |
| PXN | Paxillin | NM_002859 |
| TNS1 | Tensin 1 | NM_022648 |
| BGN | Biglycan | NM_001711 |
| FN1 | Fibronectin 1 | NM_002026 |
| ACTA2 | Actin, alpha 2, smooth muscle, aorta | NM_001613 |
| LOX1 | Lysyl oxidase | NM_002317 |
| MYH10 | Myosin, heavy chain 10, non-muscle | NM_005964 |
| TAGLN | Transgelin | NM_003186 |
| POSTN | Periostin, osteoblast specific factor | NM_006475 |
| P4HA1 | Prolyl 4-hydroxylase, alpha polypeptide I | NM_000917 |
| XYLT1 | Xylosyltransferase I | NM_022166 |
| TNC | Tenascin C | NM_002160 |
| FAP | Fibroblast activation protein, alpha | NM_004460 |
| THBS1 | Thrombospondin 1 | NM_003246 |
| NOTCH1 | Notch 1 | NM_017617 |
| JAG1 | Jagged 1 | NM_000214 |
| PDGFRB | Platelet-derived growth factor receptor, beta polypeptide | NM_002609 |
| HES1 | Hairy and enhancer of split 1, (Drosophila) | NM_005524 |
| GAPDH | Glyceraldehyde-3-phosphate dehydrogenase | NM_002046 |
| RPLP0 | Ribosomal protein, large, P0 | NM_001002 |
| HBEGF | Heparin-binding EGF-like growth factor | NM_001945 |
| TGFA | Transforming growth factor, alpha | NM_003236 |
| EGFR | Epidermal growth factor receptor | NM_005228 |
| FNDC3A | Fibronectin type III domain containing 3A | NM_014923 |
| ACTB | Actin, beta | NM_001101 |

**Additional file 3**

**Blot A**

αSMA:

**
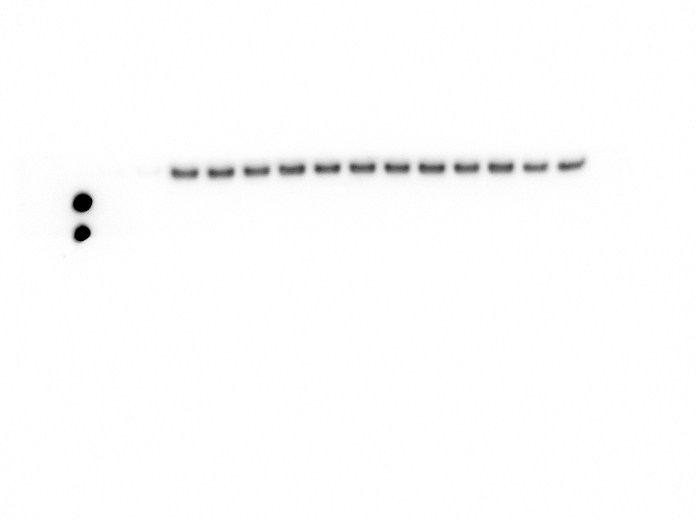
**

TubB:


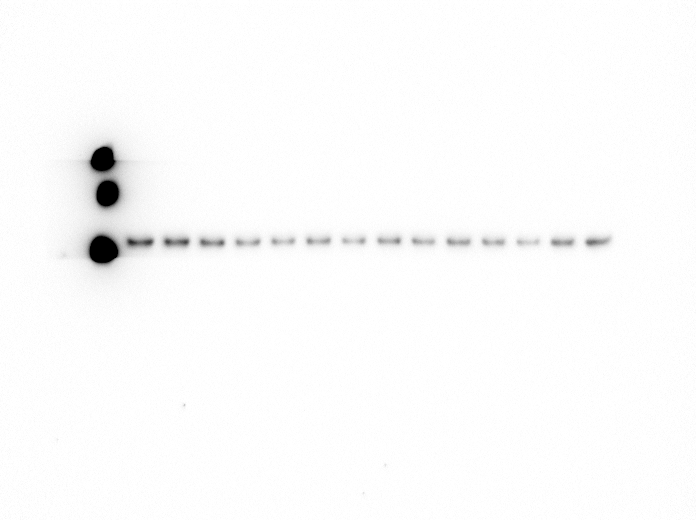


Vinc:


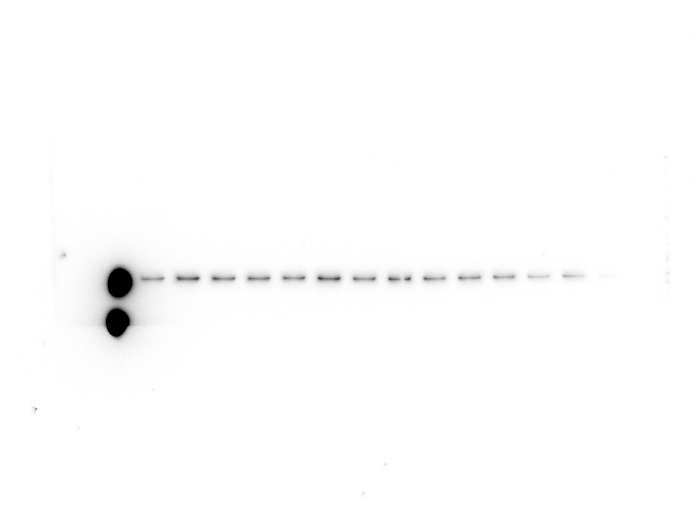


**Blot B**

MMP-14:


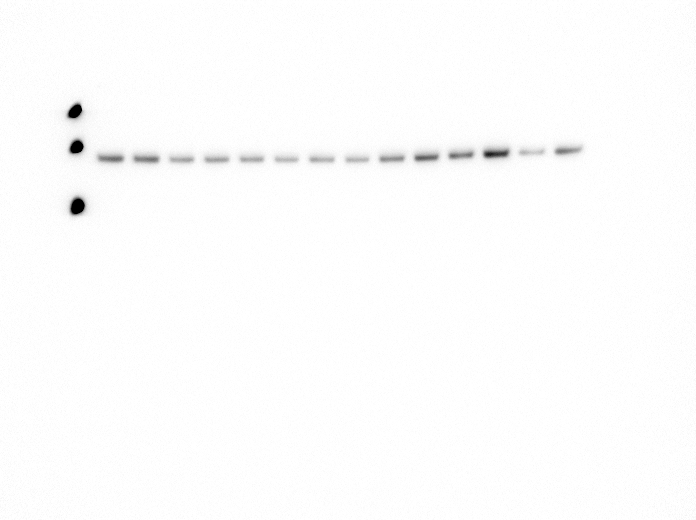


GAPDH:


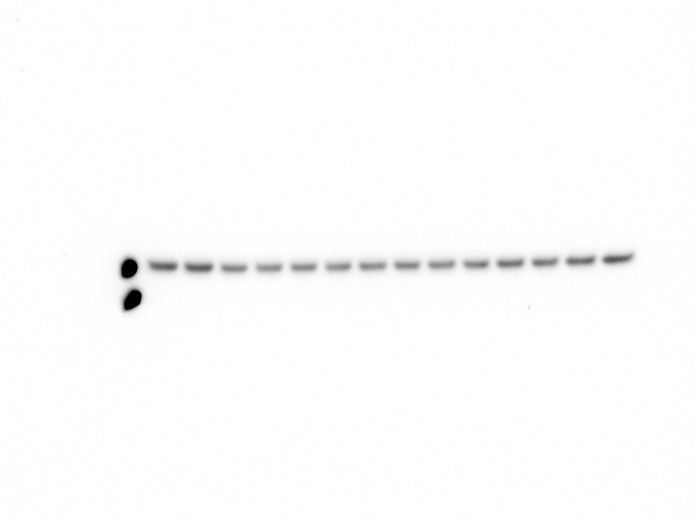


Vinc:


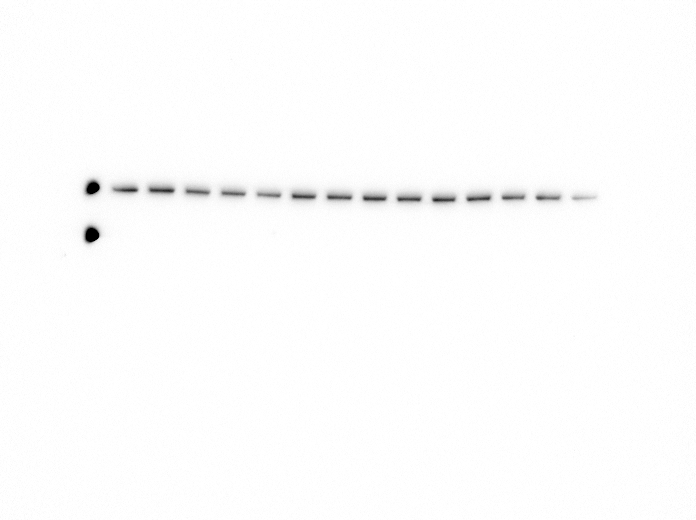


**Blot C**

MMP-2:


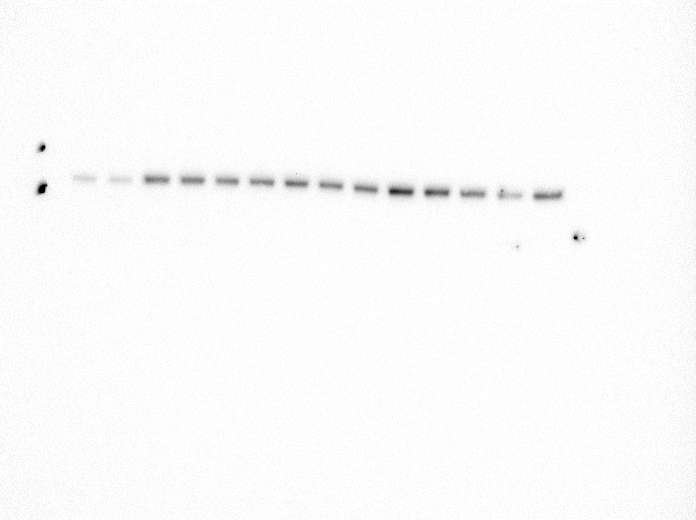


TIMP-2:


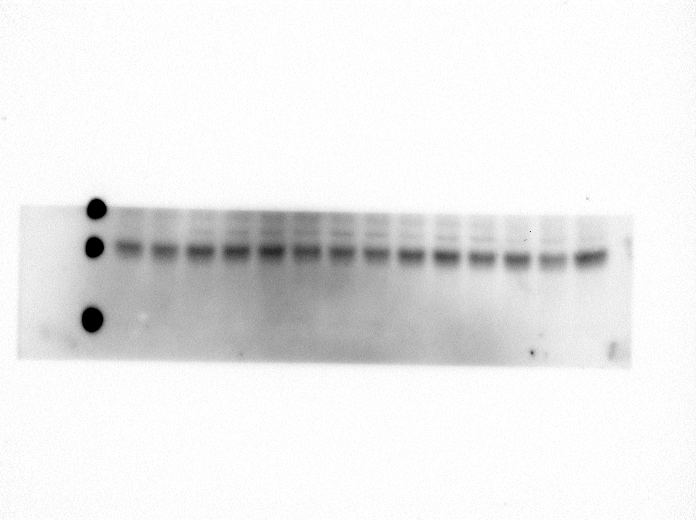


GAPDH:


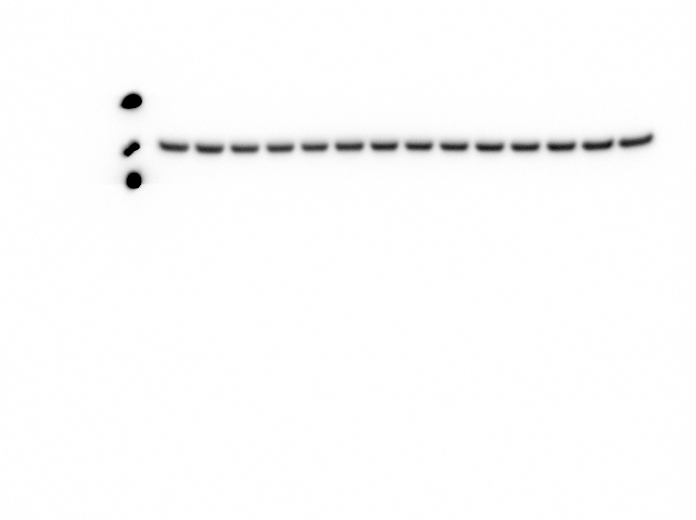


Vinc:


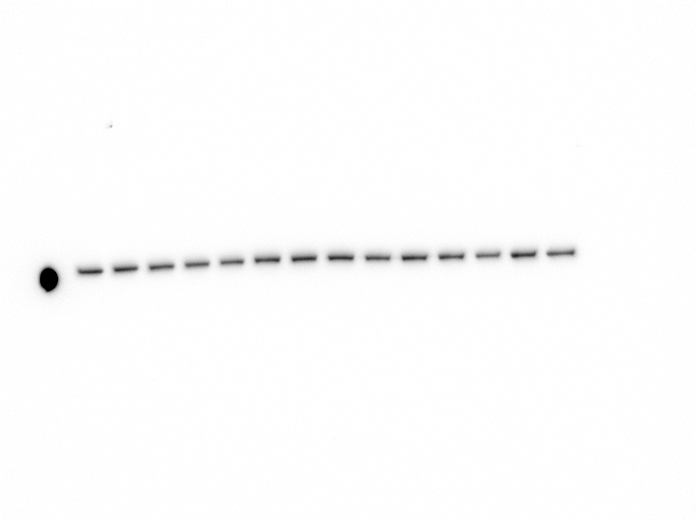


**Blot D**

TIMP-1:


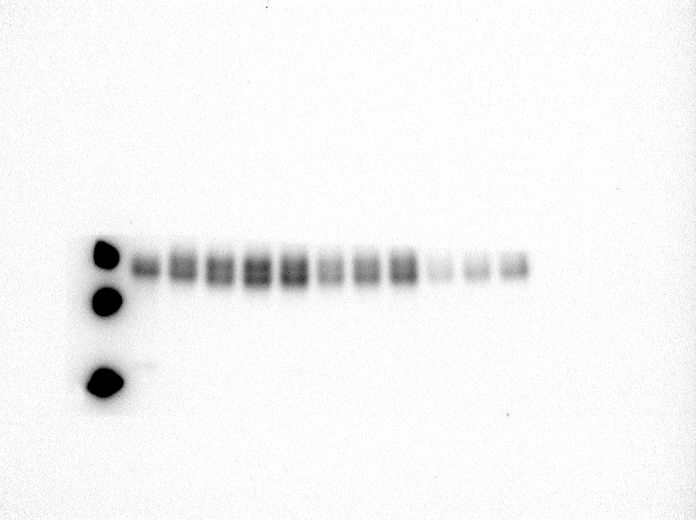


ActB:


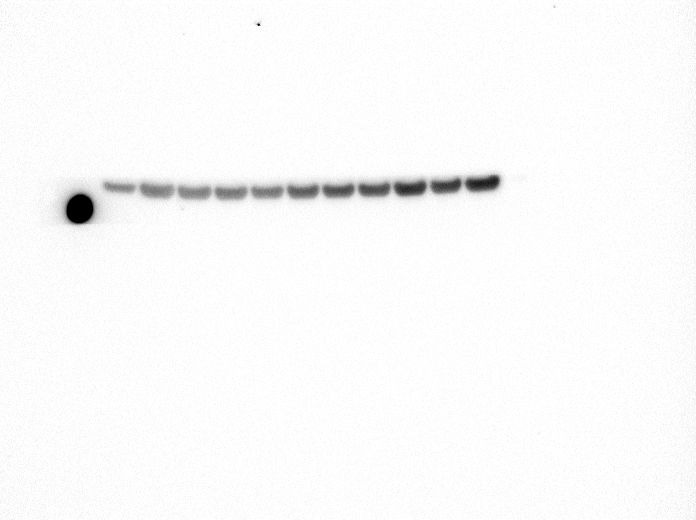


Vinc:


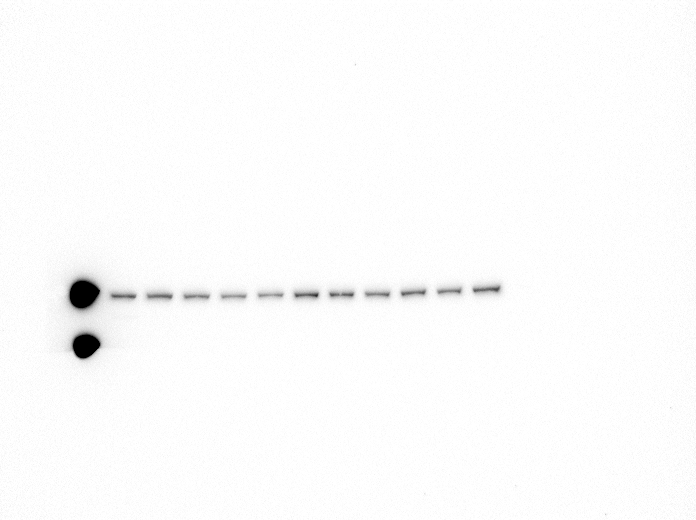


**Additional file 4**


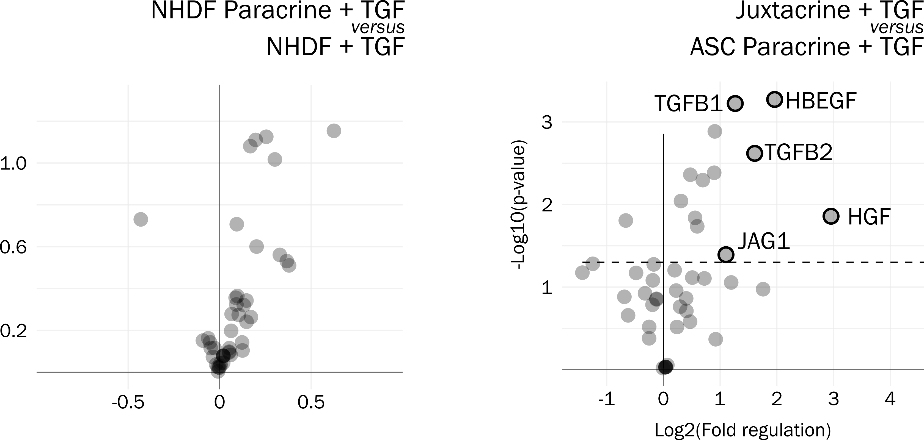


**Additional file 5**

**
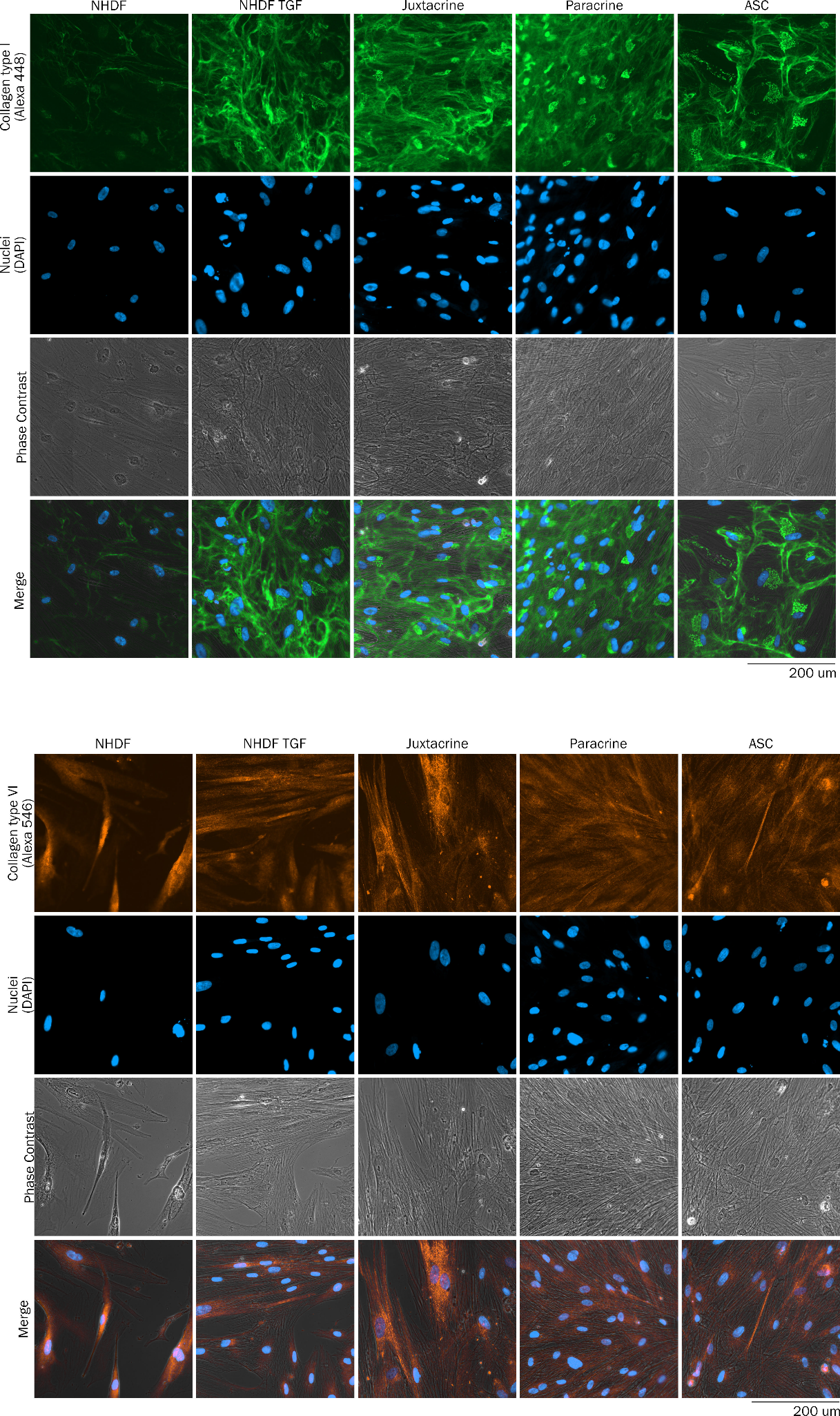
**

**Additional file 6**

**
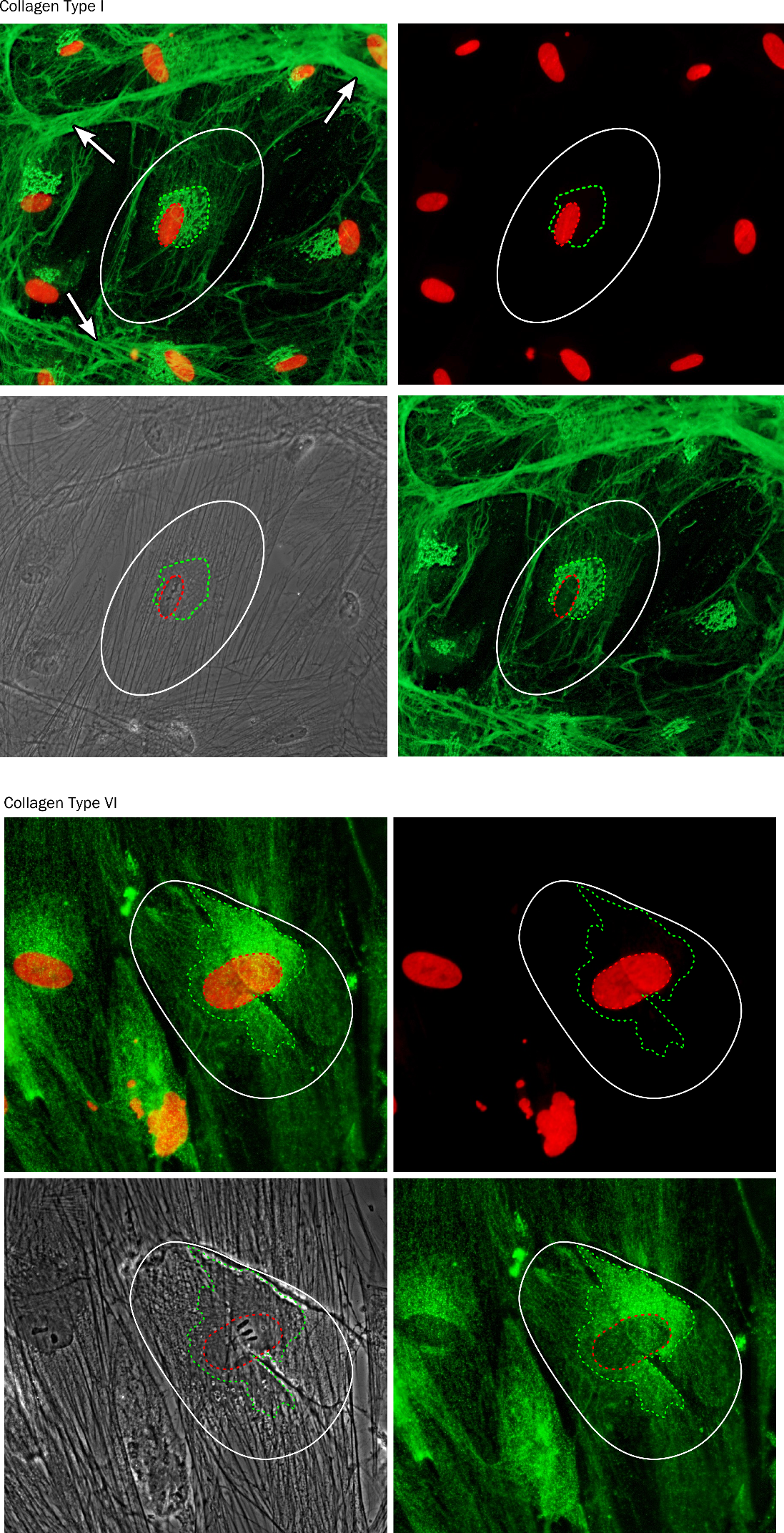
**
